# Dynamics of soil microbial communities involved in carbon cycling along three successional forests in southern China

**DOI:** 10.3389/fmicb.2023.1326057

**Published:** 2024-01-15

**Authors:** Minghui Hu, Shuyidan Zhou, Xin Xiong, Xuan Wang, Yu Sun, Ze Meng, Dafeng Hui, Jianling Li, Deqiang Zhang, Qi Deng

**Affiliations:** ^1^Key Laboratory of Vegetation Restoration and Management of Degraded Ecosystems, Guangdong Provincial Key Laboratory of Applied Botany, South China Botanical Garden, Chinese Academy of Sciences, Guangzhou, Guangdong, China; ^2^South China National Botanical Garden, Guangzhou, Guangdong, China; ^3^University of Chinese Academy of Sciences, Beijing, China; ^4^College of Life Sciences, South China Agricultural University, Guangzhou, Guangdong, China; ^5^Lushan Botanical Garden, Chinese Academy of Sciences, Jiujiang, China; ^6^Department of Biological Sciences, Tennessee State University, Nashville, TN, United States

**Keywords:** carbohydrate degradation, forest succession, metagenomics, microbial community, microbial diversity

## Abstract

Dynamics of plant communities during forest succession have been received great attention in the past decades, yet information about soil microbial communities that are involved in carbon cycling remains limited. Here we investigated soil microbial community composition and carbohydrate degradation potential using metagenomic analysis and examined their influencing factors in three successional subtropical forests in southern China. Results showed that the abundances of soil bacteria and fungi increased (*p* ≤ 0.05 for both) with forest succession in relation to both soil and litter characteristics, whereas the bacterial diversity did not change (*p* > 0.05) and the fungal diversity of Shannon-Wiener index even decreased (*p* ≤ 0.05). The abundances of microbial carbohydrate degradation functional genes of cellulase, hemicellulase, and pectinase also increased with forest succession (*p* ≤ 0.05 for all). However, the chitinase gene abundance did not change with forest succession (*p* > 0.05) and the amylase gene abundance decreased firstly in middle-succession forest and then increased in late-succession forest. Further analysis indicated that changes of functional gene abundance in cellulase, hemicellulase, and pectinase were primarily affected by soil organic carbon, soil total nitrogen, and soil moisture, whereas the variation of amylase gene abundance was well explained by soil phosphorus and litterfall. Overall, we created a metagenome profile of soil microbes in subtropical forest succession and fostered our understanding of microbially-mediated soil carbon cycling.

## 1 Introduction

Forests play an important role in the global carbon cycle, and forest soil carbon pool accounts for 73% of global soil carbon storage (more than 1,500 Pg) (Post et al., 1982; Jobbagy and Jackson, 2000). Forests at different succession stages may have different accumulation rates of soil organic carbon (Zhou et al., 2006b). Microorganisms are the direct factors driving soil organic carbon turnover and are essential to soil functions (Liang et al., 2017; Zhong et al., 2018). It has been estimated that microbial mineralization process of organic matter releases about 58 Pg carbon to atmosphere per year (Houghton, 2007). Moreover, microbial activities significantly contribute to litter decomposition, soil nutrient transformation and plant nutrient supply (Carney and Matson, 2006; van der Heijden et al., 2008; Zhu et al., 2012). Therefore, a comprehensive understanding of microbial community structure and function involved in soil carbon cycling during forest succession is crucial for better predicting of soil carbon sequestration in forest ecosystems.

During forest succession, plant types have a great impact on soil microbial community structure and diversity (Cline and Zak, 2015). The quality and quantity of organic matter input into the soil by different plant types vary with forest succession and affect the composition and diversity of the soil microbial community (Zak et al., 2003). According to ecosystem succession theory of Odum (1969), r-strategy species with faster growth rate and higher turnover rate predominate in early successional stage, while K-strategy species with a slower growth rate, lower turnover rate, and higher competitive capacity predominate in late successional stage (Yan et al., 2020). Soil microorganisms such as bacteria and fungi play major roles in soil carbon metabolism, particularly the decomposition process of soil organic carbon, such as desorption, depolymerization, and dissolution with secretory enzymes (Conant et al., 2011). Broadly speaking, fungi are considered to be K- strategist, while bacteria are considered to be r-strategist (Bardgett et al., 2005; Kaiser et al., 2014; Cheng et al., 2016). A meta-analysis of global 85 age sequences found that the fungi: bacteria ratio increased with succession (Zhou et al., 2017), suggesting that bacteria shift from r-strategist to K-strategist with the process of succession, and fungi show the opposite trend. Meanwhile, in bacterial community, phylum of *Proteobacteria* and *Bacteroidetes* are regarded as r-strategist, whereas phylum of *Acidobacteria* and *Actinobacteria* are regarded as K-strategists (Fierer et al., 2007; Zechmeister-Boltenstern et al., 2015). It has been reported that the *Acidobacteri*a and *Actinobacteria* dominated in the late successional stage (Zechmeister-Boltenstern et al., 2015), and the ratio of *Acidobacteria* + *Actinobacteria* to *Proteobacteria* + *Bacteroidetes* increased significantly with succession. However, Zhou et al. (2018) did not find a positive trend in the *Acidobacteria* + *Actinobacteria* to *Proteobacteria* + *Bacteroidetes* ratio as succession proceeds. Moreover, some studies reported that fungal community shifted from r-strategy (*Ascomycota*) to K-strategy (*Basidiomycota*) (Yan et al., 2020; Zhu et al., 2022). Moreover, some studies reported that the strategy of fungi did not show significantly change with forest succession (Ren et al., 2019; Zhong et al., 2019). Ecosystems heterogeneity may lead to differences in microbial successional patterns, and the successional pattern of microbial strategies in the subtropical high nitrogen deposition region of south China remains to be further explored.

The accumulation of soil organic carbon in successional forests is mainly the results of the action of carbon input and output, which is jointly regulated by vegetation and soil microorganisms. Soil carbon input mainly originate from litter and root exudates (Kramer et al., 2010; Clemmensen et al., 2013), while soil carbon output is mainly controlled by soil microbial community in relation to environmental factors. The changes in dominant aboveground vegetation leads to changes in soil nutrient cycling and carbon storage through their complex influence on the soil microbial community (Kelly et al., 2021). Previous studies were mainly concentrated on the variation of soil microbial community structure in successional forests (Sokolowska et al., 2020; Jiang et al., 2021; Shang et al., 2021; Wang et al., 2022), whereas the succession pattern of soil carbohydrate metabolism by microorganisms at the genetic level have received little attention. In general, soil organic carbon degradation is a microbial process driven by functional genes participating in carbohydrate metabolism. Difference in the expression of specific carbon degradation genes in different ecosystems determine nutrient availability, organic carbon turnover, and ultimate carbon retention in soil (Trivedi et al., 2016; Kelly et al., 2021). Exploring the variation of microbial functional genes in successional forests can provide a more intuitive understanding of microbially-mediated soil organic carbon turnover.

In this study, the composition and diversity of soil microbial communities as well as their potential functional changes were studied using a high-throughput sequencing technology in three representative forest types of the successional sequences in the south subtropical region of China. We aimed to determine: (1) whether the composition and diversity of soil microbial communities varied along the forest succession; (2) and what were the patterns of soil microbial functional potential of carbohydrate degradation shifts; (3) and what factors influence the microbial functional potential of carbohydrate degradation.

## 2 Materials and methods

### 2.1 Study sites

The study was conducted at Dinghushan Biosphere Reserve in Guangdong Province, China (23°09′21″-23°11′30″ N, 112°30′39″-112°33′41″ E). The area of this region covers approximately 1,133 ha of forests. The annual average temperature is 20.9°C, with the highest and lowest temperatures of 38°C and −0.2°C, respectively. The annual rainfall in this area is 1,950 mm, and the main rainfall season is from April to September, accounting for approximately 70% of the annual rainfall. The mean annual relative humidity is 80.3%. The forest coverage rate of Dinghushan Biosphere Reserve reaches 98.7%. The three types of successional forests studied in this experiment are as follows: (1) *Pinus massoniana* forest (approximately 70 years old, PMF). The dominant species is *P. massoniana*, and the canopy density is approximately 50%; (2) Pine and broadleaf mixed forest (approximately 100 years old, PBMF). The dominant species are *P. massoniana*, *Schima superba*, and *Castanopsis chinensi*, and the canopy density is approximately 90%; and (3) Monsoon evergreen broadleaf forest (approximately 400 years old, MEBF). The dominant species are *C. chinensis*, *Cryptocarya concinna*, *S. superba*, and *Machilus chinensis*, and the canopy density is approximately 95%. These three types of forests represent the early, middle, and late succession of the Dinghushan forest ecosystem, respectively.

### 2.2 Soil and litter sampling

The experiment and soil sampling were initiated in August 2019. Before sampling, we removed humus, litter, and other impurities from the topsoil. Five scattered soil samples were collected from 0–10 cm soil layer and mixed into one sample each time using a soil auger (5 cm inner diameter). Five replications were collected in each forest, obtaining 15 soil samples in total. All soil samples were screened with a 2-mm sieve to remove roots and gravel. Each soil sample was divided into two parts; one part was stored in the freezer at −80°C for subsequent soil metagenomic determination, and the other was air-dried for measuring soil physicochemical properties. The litterfall of three successional forests was collected in 1 m × 1 m collection frame, and we recorded the total dry weight in each frame.

### 2.3 Soil and litter properties measurements

The content of soil organic carbon (SOC) and total nitrogen (TN) were determined using a high sample throughput automatic elemental analyzer (Vario max cube, Elementar, Germany). The content of total phosphorus (TP) was determined using sulfuric-perchloric acid digestion and molybdenum-antimony colorimetry. The pH of soil samples was determined using potentiometric method, and the ratio of water to soil was 1:2.5. Soil readily oxidized organic carbon (ROC) was defined as the organic carbon that can be oxidized by a potassium permanganate (KMnO_4_) solution with a concentration of 333 mMol/L, and was determined using Blair et al.’s (1995) method. Soil dissolved organic carbon (DOC) was determined using Marten’s (1995) method. Soil temperature and moisture was determined by Time domain reflectometry (TDR, Campbell, USA). Litterfall (plant dead organic material such as leaves, branches, fruit (flower) drops, bark, and mosses and lichens) data comes from Dinghushan Forest Ecosystem Research Station, in which litter carbon content was determined by Total Carbon Analysis (TOC-VCPH SHIMADZU, Japan) and litter nitrogen content was analyzed by Kjeldahl method.

### 2.4 DNA extraction, library construction, and metagenomic sequencing

Total genomic DNA was extracted from 0.5 g of soil samples using the FastDNA® Spin Kit for Soil (MP Biomedicals, Norcross, GA, USA) according to the manufacturer’s instructions. The concentration and purity of extracted DNA were determined by TBS-380 (Turner BioSystems Inc., USA) and NanoDrop2000 (Thermo Fisher Scientific, USA), respectively. DNA extract quality was detected on 1% agarose gel.

The average size of extracted DNA samples were sonicated [Covaris M220 (Gene Company Limited, China)] resulting in an average size of 400 bp and these were used to construct the paired-end library. The paired-end library was constructed using NEXTFLEX Rapid DNA-Seq (Bioo Scientific, Austin, TX, USA). Adapters containing the full complement of sequencing primer hybridisation sites were connected to the blunt-end fragments. A dual indexed barcoding information for sample identification and differentiation was incorporated into the library preparation process. Paired-end sequencing was performed on Illumina NovaSeq (Illumina Inc., San Diego, CA, USA) at Majorbio Bio-Pharm Technology (Shanghai, China) using NovaSeq Reagent Kits according to the manufacturer’s instructions. The sequencing produced paired-end reads of 150 bp. All sequences associated with this project have been submitted in the NCBI Short Read Archive database (Accession Number: PRJNA782859).

### 2.5 Genome assembly, construction of non-redundant gene catalog, and functional annotation

The adapter sequences of paired-end Illumina reads were trimmed using fastp (Chen et al., 2020).^1^ For ensuring the quality of our metagenomic data, we processed the raw reads using a 5-bp sliding window, trimming sequences with a quality score lower than Q20. This threshold, indicative of a read accuracy of at least 99%, was employed to eliminate low-quality sequences and retain high-quality pair-end and single-end reads for subsequent analyses. The reads with a length of less than 50 bp after trimming process were removed from further analysis. Metagenomics data were assembled using MEGAHIT (Li et al., 2015)^2^ based on the principle of the succinct de Bruijn graphs (Supplementary Table S2). Since contigs shorter than 300 bp are often considered less reliable for providing accurate genomic insights due to the limited sequence context they offer (Sameith et al., 2017), only contigs with a length of or over 300 bp were selected as the final assembling result and used for further gene prediction and annotation. Open reading frames (ORFs) prediction of each assembled contig was performed using MetaGene (Noguchi et al., 2006).^3^ The predicted ORFs with a length of or over 100 bp were selected and translated into amino acid sequences using the National Center for Biotechnology Information (NCBI) translation table.

CD-HIT (Fu et al., 2012)^4^ was used to construct a non-redundant gene catalog (parameters: 90% sequence identity and 90% coverage). High-quality reads were mapped to the non-redundant gene catalog with 95% identity using SOAPaligner (Li et al., 2008),^5^ and gene abundance in each sample was evaluated. The representative sequences of non-redundant gene catalog were aligned with the NCBI NR database for taxonomic annotations using Diamond (Buchfink et al., 2015),^6^ and the cut-off e-value was 1e^−5^. The KEGG annotation was performed using the Kyoto Encyclopedia of Genes and Genomes database^7^ using Diamond (Buchfink et al., 2015) (see Footnote 6) with an e-value cut-off of 1e^−5^.

### 2.6 Statistical analyses

The species information about bacteria and fungi were selected from the NR database, while the functional genes were selected from the KEGG database (Supplementary Tables S4–S6). Reads per kilobase per million mapped reads (RPKM) values were employed to calculate alpha diversity and all gene abundance values and compared between different samples; the gene abundance values in replicated samples were averaged.

One-way analysis of variances and Duncan’s multiple range test were conducted using IBM SPSS 21.0 (IBM Corporation, Armonk, NY, USA) to explore the differences in soil physicochemical properties and functional genes abundance. Chao1 index, Shannon–Wiener index, and Simpson index was used to evaluate microbial α-diversity in successional forests, and the significant differences were set at *p* ≤ 0.05.

To visualize the differences of microbial community composition and functional genes’ abundance, principal coordinate analysis (PCoA) based on Bray–Curtis distance was employed (R vegan package v2.5-6) in R v4.1.0. Analysis of similarities (ANOSIM) based on Bray–Curtis distance was performed to test the differences (microbial community composition and functional genes’ abundance) between groups and the differences within groups (R vegan package v2.5-6). Mantel test results were used to explore correlations among environmental factors and microbial community and the microbial carbohydrate degradation genes based on the Spearman correlation coefficient in R v4.1.0. Correlation heatmap with signs was performed using R v4.1.0. Metabolic pathways are based on information from KEGG database,^8^ we use the Spearman correlation coefficient to specific environmental factors in the process of metabolic pathways. Random Forest models were used to identified the most important environmental variables, and the importance of variables was evaluated by classifying multiple decision tree (Breiman, 2001). The analyses were conducted using the randomForest package (Liaw and Wiener, 2002) in R v4.1.0. The A3 package were used to evaluate the significance of the model and the cross-validation *R*^2^, and the rfPermute package was used to assess the importance of each predictor to soil carbohydrate degradation functional genes.

Distance-based redundancy analysis (db-RDA) was used to evaluate the effects of soil physicochemical properties on microbial functional genes’ abundance (R vegan package v2.5-6). We calculated the β-diversity of species and functional genes using linear regression analysis to assess the conformance between species and functional genes, respectively (R vegan package v2.5-6). Further, to quantify the functional contribution of specific phylums and species, the correlation analysis between species and function relative abundance was conducted based on the corresponding relationship between species and functions in the samples (R vegan package v2.5-6).

## 3 Results

### 3.1 Soil physicochemical and litter characteristics

With forest succession, the soil physicochemical properties and litter characteristics showed different changing patterns (Table 1). The soil physicochemical properties varied significant among the three forest types (Table 1). SOC, DOC, NROC, TN, and SM showed an upward trend with forest succession (*p* ≤ 0.05 for all). Compared with PMF, TP and soil pH significantly increased in PBMF but decreased in MEBF (Table 1). The litter characteristics changed significantly as well (Table 1). Litterfall decreased significantly in PBMF and increased in MEBF compared to PMF. The highest litter C/N were observed in the PMF.

**Table 1:** Soil physicochemical properties in successional forests.

Property	PMF	PBMF	MEBF
SOC (g/kg)	22.41 ± 3.08 (a)	28.06 ± 1.53 (b)	35.04 ± 2.85 (c)
DOC (g/kg)	0.55 ± 0.1 (a)	0.64 ± 0.11 (ab)	0.74 ± 0.14 (b)
ROC (g/kg)	11.85 ± 0.42	11.65 ± 0.64	12.32 ± 0.29
ROC/SOC	0.54 ± 0.07 (c)	0.42 ± 0.01 (b)	0.35 ± 0.02 (a)
TN (g/kg)	1.47 ± 0.14 (a)	2.04 ± 0.12 (b)	2.71 ± 0.17 (c)
TP (g/kg)	0.22 ± 0.02 (a)	0.41 ± 0.04 (c)	0.31 ± 0.04 (b)
pH	3.91 ± 0.09 (a)	4.06 ± 0.09 (b)	3.97 ± 0.07 (ab)
SM (% Vol.)	0.21 ± 0.02 (a)	0.31 ± 0.02 (b)	0.46 ± 0.05 (c)
ST (°C)	25.98 ± 0.36	26.12 ± 0.31	25.96 ± 0.34
Litterfall (g/m^2^/yr)	1083.24 ± 36.11 (c)	661.05 ± 47.44 (a)	788.19 ± 46.14 (b)
Litter C/N	40.2 ± 7 (b)	28.68 ± 3.12 (a)	24.37 ± 2.42 (a)

PMF, *Pinus massoniana* forest; PBMF, pine and broadleaf mixed forest; MEBF, monsoon evergreen broadleaf forest; SOC, total soil organic carbon; DOC, dissolved organic carbon; ROC, readily oxidized organic carbon; TN, total nitrogen; TP, total phosphorus; SM, soil moisture; ST, soil temperature; Litter C/N, the ratio of litter C to litter N. Mean values ± standard deviations are given (*n* = 5). Different lowercase letters (in brackets) indicate a significant difference (*p* ≤ 0.05) among the forest types.

### 3.2 Soil microbial community composition

**Figure 1:**
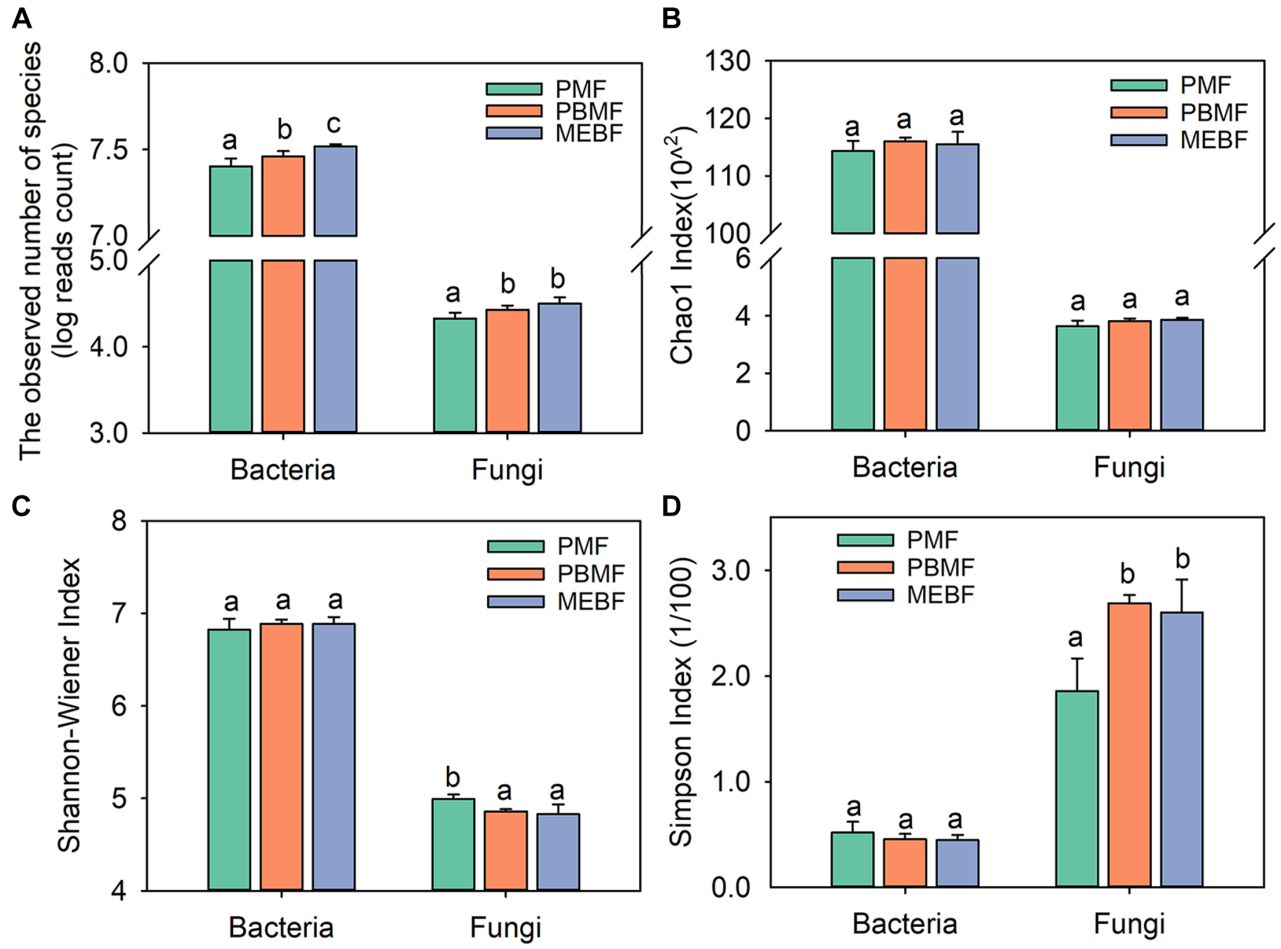
Relative abundances of soil bacterial and fungal based on reads counts **(A)**, derived from sequence alignment using SOAPaligner, Chao1 index **(B)**, Shannon–Wiener index **(C)**, and Simpson index **(D)** in successional forests. The values are the means of five replicates (± standard deviations), and different lowercase letters represent significant differences. PMF, *Pinus massoniana* forest; PBMF, pine and broadleaf mixed forest; MEBF, monsoon evergreen broadleaf forest.

Taxonomic annotation information of sample species was obtained and compared with the NR database. A total of eight kingdoms, 135 phyla, 292 classes, 683 orders, 1,295 families, 3,275 genera, and 17,379 species were detected in PMF, PBMF, and MEBF. Both soil bacterial and fungal abundances increased significantly along the gradient of forest succession (Figure 1A, *p* ≤ 0.05 for both).

**Figure 2:**
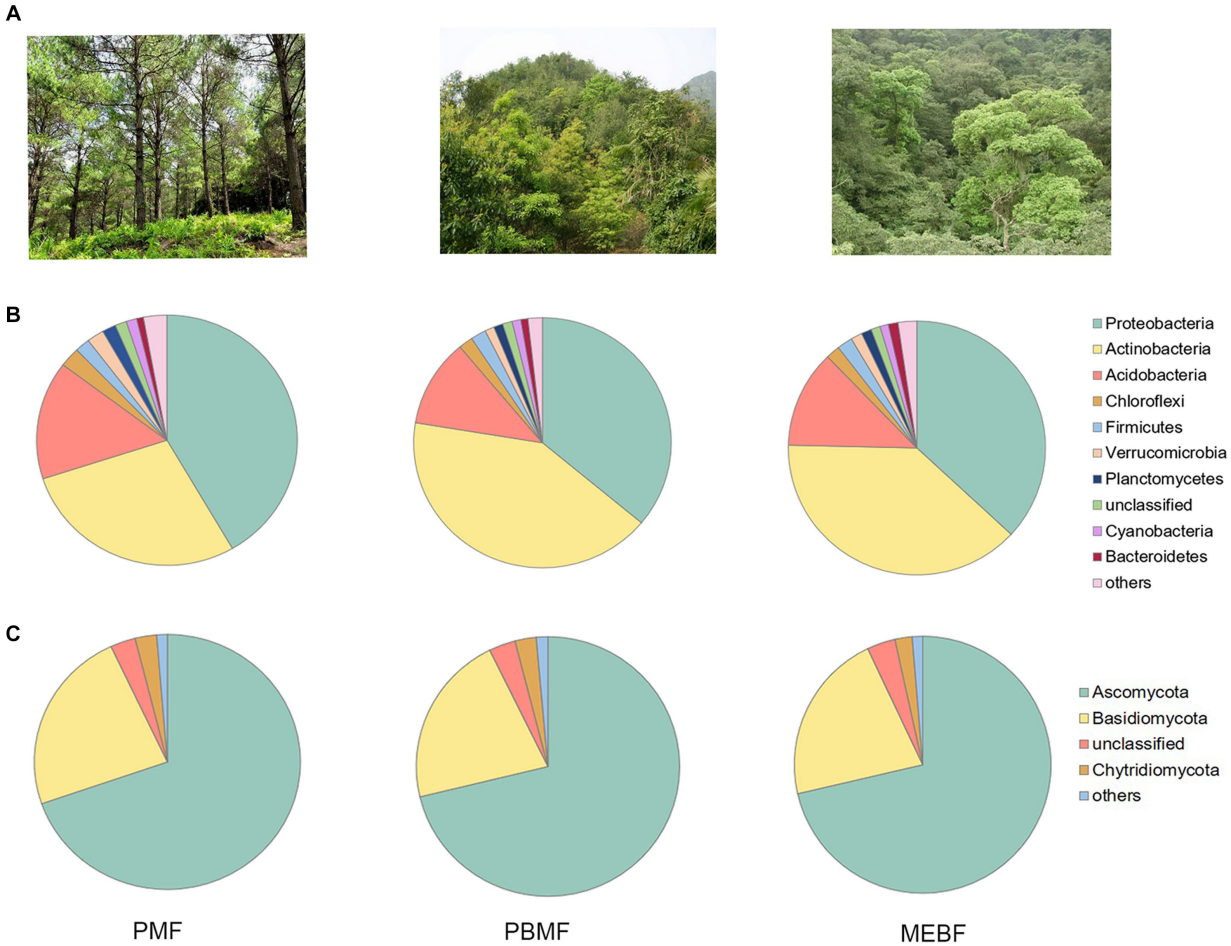
Photographs of each stage of successional forests **(A)**. Composition of major taxa of bacteria **(B)** and fungi **(C)** at the phylum level in successional forests. PMF, *Pinus massoniana* forest; PBMF, pine and broadleaf mixed forest; MEBF, monsoon evergreen broadleaf forest.

The Chao1 index, Shannon–Wiener index, and Simpson index of bacterial community showed no significant difference with succession (Figures 1B–D; *p* > 0.05 for all). The Chao1 index of fungal community also did not differ among the three forests (Figure 1B). The Shannon–Wiener index of fungal community decreased significantly with succession (Figure 1C; *p* ≤ 0.05), while the Simpson index increased significantly (Figure 1D; *p* ≤ 0.05). The photographs of each stage of successional forests are shown in Figure 2A. At the phylum level, soil bacterial communities were mainly composed of *Proteobacteria* (36.06–41.63%), *Actinobacteria* (28.38–41.51%), and *Acidobacteria* (28.38–41.51%) during all stages (Figure 2B; Supplementary Table S1). Venn diagrams showed that 11,938 species of bacteria and 402 species of fungi were shared in the three successional forests, respectively (Supplementary Figure S2). Soil fungal communities were mainly composed of *Ascomycota* (69.69–71.38%) and *Basidiomycota* (21.45–23.35%) (Figure 2C; Supplementary Table S1).

**Figure 3:**
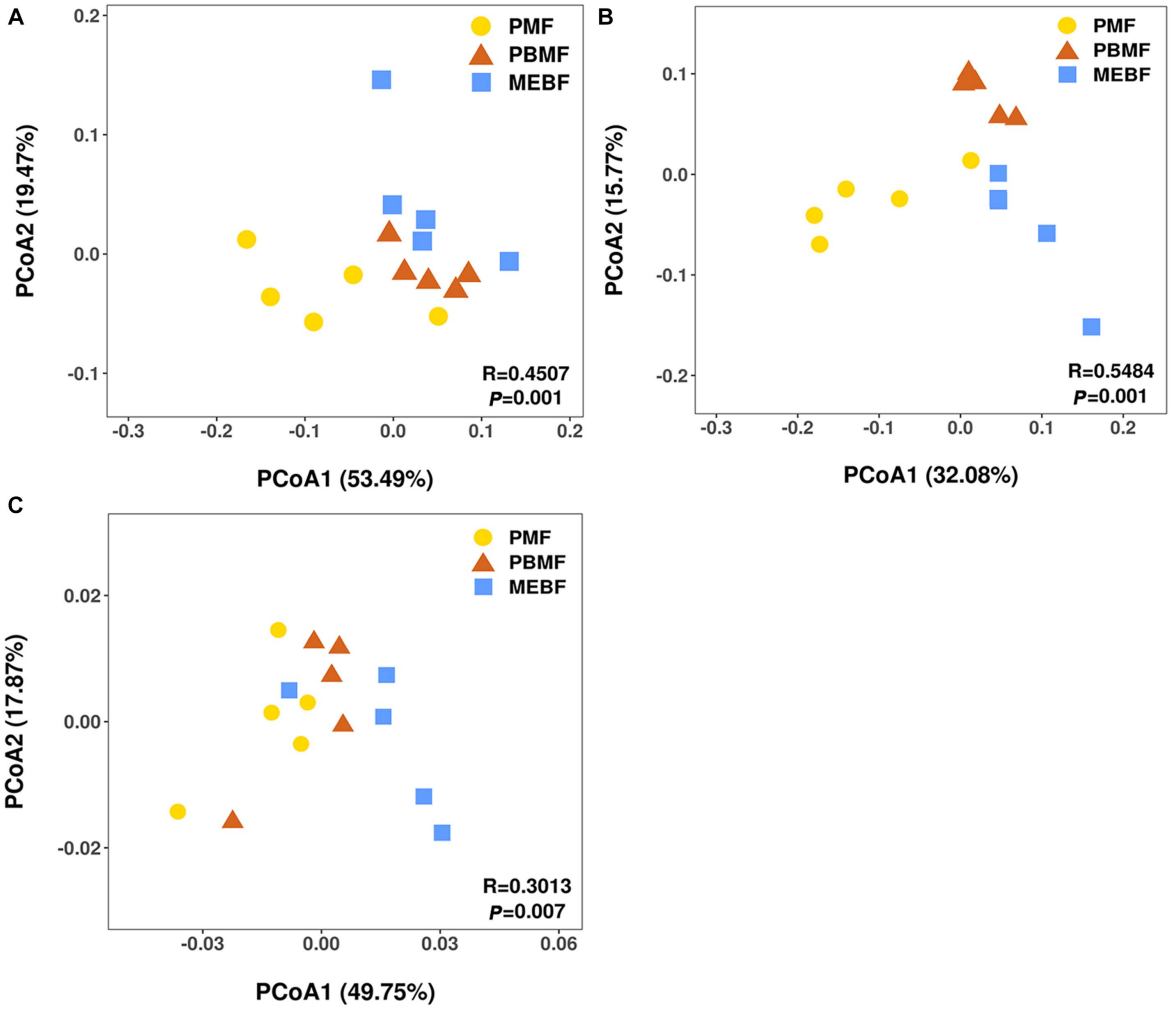
Principal coordinate analysis (PCoA) based on the Bray–Curtis distance of samples for bacterial **(A)** and fungal **(B)** communities, carbohydrate degradation gene composition **(C)** in successional forests. PMF, *Pinus massoniana* forest; PBMF, pine and broadleaf mixed forest; MEBF, monsoon evergreen broadleaf forest.

The ANOSIM analysis revealed that the differences between bacterial and fungal communities in successional forests were significantly greater than those within the groups (Supplementary Figures S1A,B). The PCoA results showed significant differences among bacterial and fungal communities (Figures 3A,B), which were related to soil physicochemical properties and litter characteristics (Supplementary Figures S3A,B; Table 2).

**Table 2:** Mantel test results based on the Bray–Curtis distance that were used to explore correlations among carbohydrate degradation genes, bacterial communities, fungal communities, soil physicochemical characteristics (SOC, DOC, ROC, TN, TP, pH, SM, and ST), and litter characteristics (litterfall and litter C/N).

	Soil characteristics	Litter characteristics	Bacterial communities	Fungal communities	Carbohydrate degradation genes
Soil characteristics	1				
Litter characteristics	0.3466**	1			
Bacterial communities	0.3104*	0.3002*	1		
Fungal communities	0.3836*	0.2977**	0.8471***	1	
Carbohydrate degradation genes	0.4465**	0.3357*	0.2878*	0.3605**	1

*Indicates *p* ≤ 0.05; **indicates *p* ≤ 0.01, ***indicates *p* ≤ 0.001.

### 3.3 Microbial genes involved in soil carbohydrate decomposition

**Figure 4:**
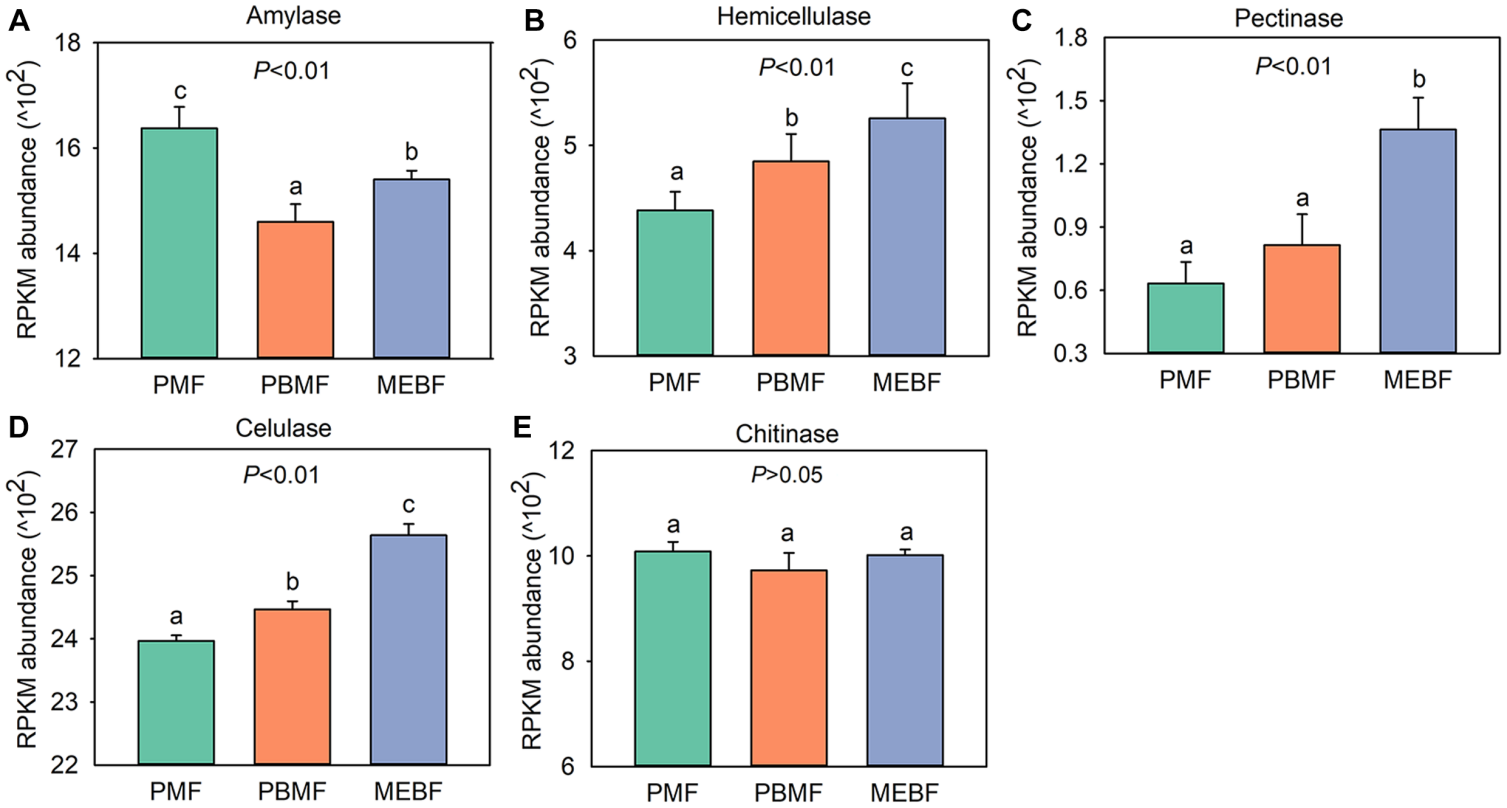
Abundance of functional genes in soil carbohydrate degradation in successional forests. **(A)** Amylase; **(B)** hemicellulose; **(C)** pectinase; **(D)** cellulase; **(E)** chitinase. RPKM values are calculated as the number of reads mapped to a gene per kilobase of its transcript length, scaled to a million mapped reads for each sample. The data are means ± standard deviations, and *n* = 5. Different lowercase letters represent significant differences among forest types. PMF, *Pinus massoniana* forest; PBMF, pine and broadleaf mixed forest; MEBF, monsoon evergreen broadleaf forest.

Using the KEGG database to obtain the KEGG annotation profile corresponding to the genes present in the samples, we found a total of 9,885 KEGG orthologous genes from PMF, PBMF, and MEBF. Soil microbial functional genes related to degradation of soil carbohydrates were selected from the KEGG database (Supplementary Table S3). PCoA results illustrated that the functional potential in soil carbohydrate degradation was statistically significantly different among the three forests (Figure 3C). As shown in Figure 4, the gene abundance of amylase was highest in PMF and lowest in PBMF (Figure 4A). The gene abundance of pectinase did not change significantly in PBMF, but increased significantly in MEBF (Figure 4C). The gene abundance of hemicellulase and cellulase increased significantly in each stage of succession (Figures 4B,D). However, the gene abundance for chitinase showed no significantly difference with succession (Figure 4E).

**Figure 5:**
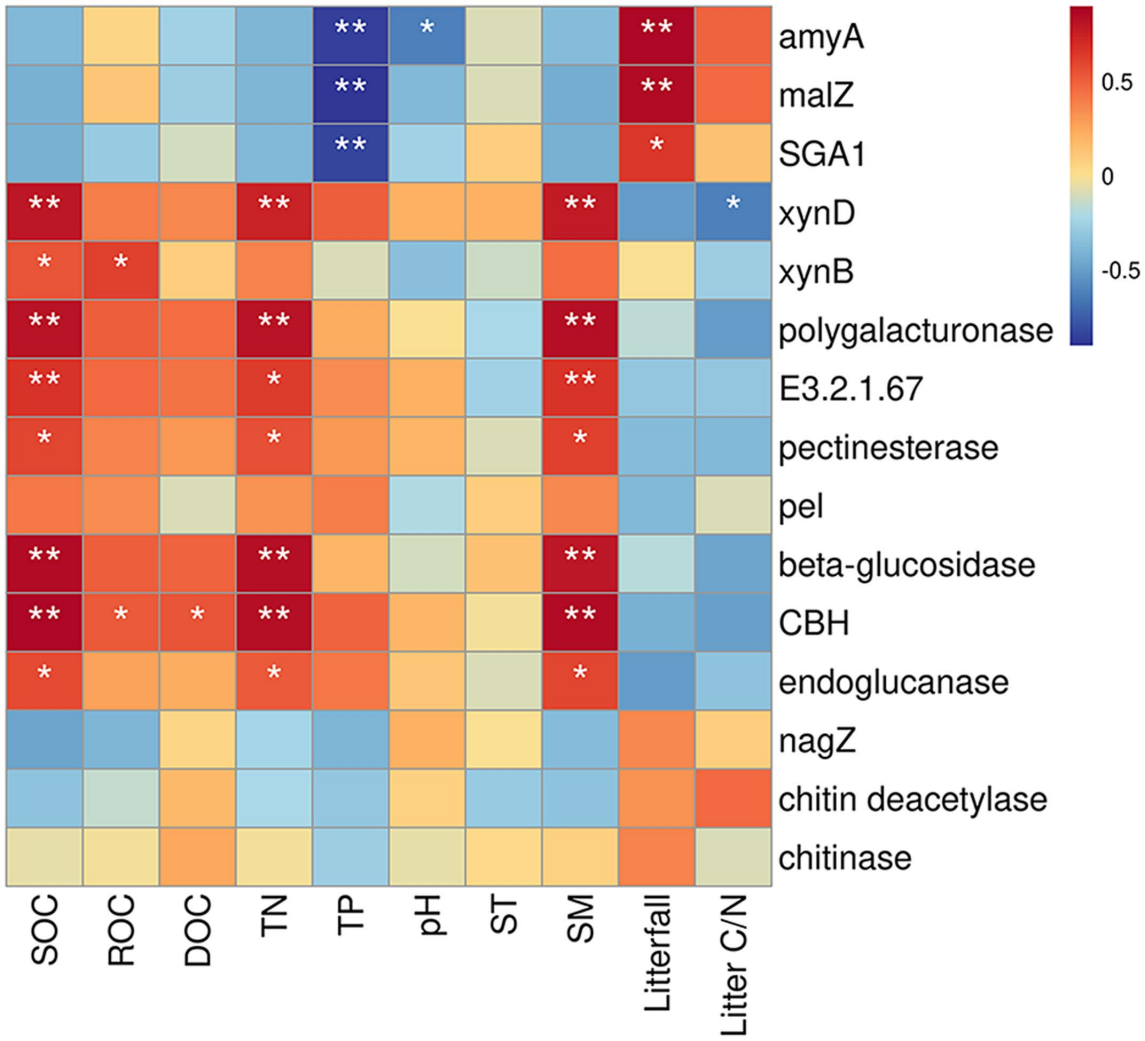
Correlation between environmental factors and soil carbohydrate degradation genes based on the Spearman correlation coefficient. The color gradients represented Spearman correlation coefficient: red indicates positive correlation, and blue indicates negative correlation. *Indicates *p* ≤ 0.05; ** indicates *p* ≤ 0.01.

**Figure 6:**
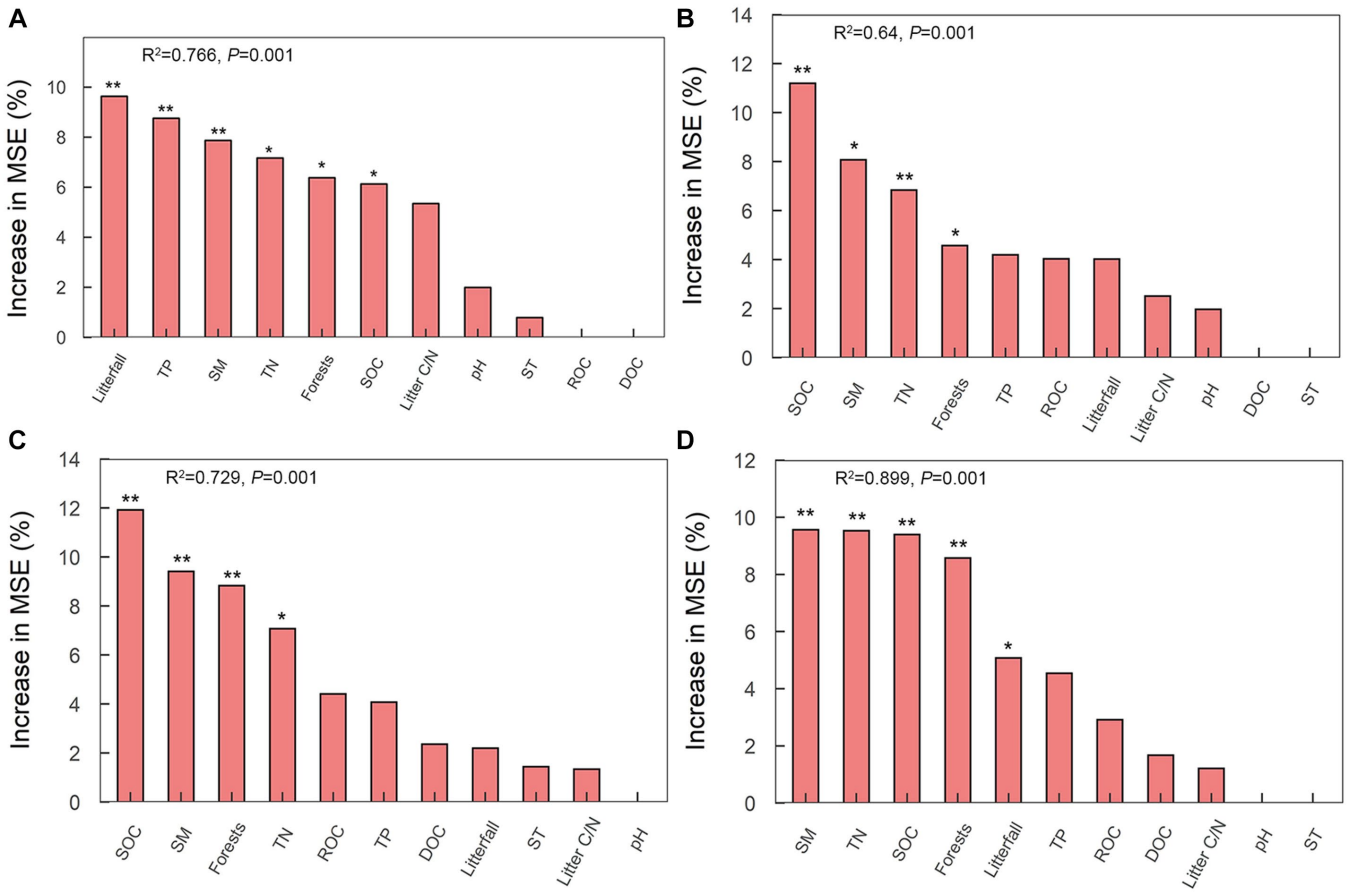
Random forest model of relationships between environmental factors and soil carbohydrate degradation genes in successional forests. The model shows the average predictive importance (mean square error (MSE) increase percentage) for each environmental factor for soil carbohydrate degradation genes. **(A)** Amylase; **(B)** hemicellulase; **(C)** pectinase; **(D)** cellulase. *Indicates *p* ≤ 0.05; ** indicates *p* ≤ 0.01.

**Figure 7:**
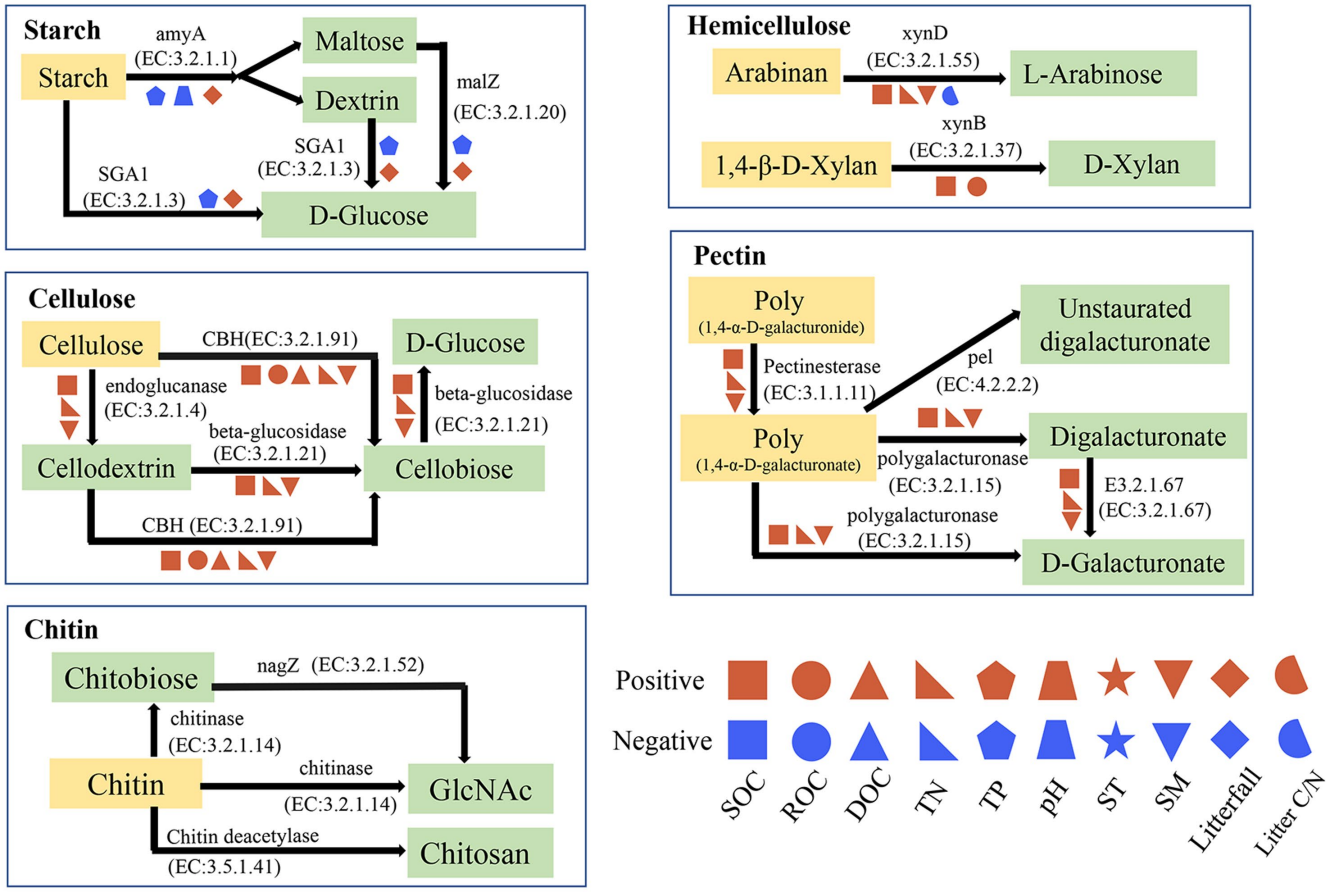
Metabolic pathway of soil carbohydrate degradation and related environmental factors. SOC, total soil organic carbon; DOC, dissolved organic carbon; ROC, readily oxidized organic carbon; TN, total nitrogen; TP, total phosphorus; SM, soil moisture; ST, soil temperature; Litter C/N, the ratio of litter C to litter N. The *p* values less than or equal to 0.05 are presented.

Fifteen specific genes, coding for amylase, pectinase, hemicellulase, cellulase and chitinase, responded significantly to soil physicochemical and litter characteristics (Figure 5). There were significant correlations between the relative abundance of genes encoding amylase (amyA, malZ, and SGA1) and TP (*n* = 3 genes), pH (*n* = 1 gene), and litterfall (*n* = 3 genes) (Figure 5). The Random Forest model suggested that litterfall, TP, SM, TN, SOC and litter C/N were the most important predictors of amylase gene in successional forests (Overall model: *R*^2^ = 0.766, *p* = 0.001; environment variable *p* ≤ 0.05, Figure 6A). The relative abundance of genes encoding hemicellulase (xynD, xynB) were significantly correlated with SOC (*n* = 2 genes), ROC (*n* = 1 gene), TN (*n* = 1 gene), SM (*n* = 1 gene) and litterfall (*n* = 1 gene) (Figure 5). The Random Forest model suggested that SOC, SM and TN were the most important predictors of hemicellulase gene in successional forests (Overall model: *R^2^* = 0.64, *p* = 0.001; environment variable *p* ≤ 0.05, Figure 6B). The relative abundance of genes encoding pectinase (Polygalacturonase, E3.2.1.67 and pectinesterase) were significantly correlated with SOC (*n* = 3 genes), TN (*n* = 3 genes) and SM (*n* = 3 genes) (Figure 5). The Random Forest model suggested that SOC, SM and TN were the most important predictors of pectinase gene in successional forests (Overall model: *R*^2^ = 0.729, *p* = 0.001; environment variable *p* ≤ 0.05, Figure 6C). The relative abundance of genes encoding cellulase (beta-glucosidase, CBH and endoglucanase) were significantly correlated with SOC (*n* = 3 genes), ROC (*n* = 1 gene), DOC (*n* = 1 gene), TN (*n* = 3 genes) and SM (*n* = 3 genes) (Figure 5). The Random Forest model suggested that SOC, SM, TN and litterfall were the most important predictors of cellulase gene in successional forests (Overall model: *R*^2^ = 0.899, *p* = 0.001; environment variable *p* ≤ 0.05, Figure 6D). The metabolic pathway of soil carbohydrate degradation and related environmental factors are shown in Figure 7.

### 3.4 Link between soil carbohydrate degradation and functional genes

To evaluate the consistency between microbial communities and functions, we performed a linear regression analysis using Bray-Curtis distance based on the β-diversity of microbial communities and functions. We observed a significant and positive linear relationship, which indicates that the change of microbial community composition will change the functional composition (*R*^2^ = 0.67, *p* = 0.001, Supplementary Figure S4A).

To visualize the association between species and functions in the samples, we mapped species (in phylum and species level) to functional genes to discover the contribution of species to specific functions. Functional genes encoding for carbohydrate degradation were clustered at level 2 on KEGG (Supplementary Figure S4A). *Actinobacteria*, *Proteobacteria* and *Acidobacteria* were the main contributor of carbohydrate degradation in microbial communities across successional forests (Supplementary Figure S4). As it shown in Supplementary Figure S5, at species level, *Thermomonospora curvata* was the main contributor of starch degradation, accounting for 2.71% ~ 3.95%; *Silvibacterium bohemicum* was the main contributor of hemicellulose degradation, accounting for 9.5% ~11.42%; *Actinospica robiniae* was the main contributor of hemicellulose degradation, accounting for 4.64% ~ 6.37%; *Terracidiphilus gabretensis* was the main contributor of hemicellulose degradation, accounting for 16.72% ~ 21.6%; *Candidatus Koribacter versatilis* was the main contributor of hemicellulose degradation, accounting for 3.93% ~ 9.38%. As for individual carbohydrate degradation genes, *Terracidiphilus gabretensis* was the main contributor of SGA1, beta-glucosidase, endoglucanase and polygalacturonase, accounting for 3.06% ~ 7.46, 4.23% ~ 5.31, 3.31% ~ 4.19, and 21.58% ~26.58%. *Silvibacterium_bohemicum* was the main contributor of xynB and chitinase.

## 4 Discussion

### 4.1 Changes in microbial community structure

The typical forest types at three different succession stages in Dinghushan Biosphere Reserve provide a unique dynamic landscape for studying the succession pattern of microbial community structure and their influencing factors. The continuous increase in bacterial and fungal biomass with forest succession can be attributed in part to the increased availability of soil nutrients (Supplementary Figure S3; Table 2). Both soil physicochemical and litter characteristics were closely associated with soil bacterial and fungal community structure (Supplementary Figure S3; Table 2). During forest succession, due to changes in plant community types, especially in the composition of dominant tree species, litter characteristics and soil physicochemical changed correspondingly (Wedin and Tilman, 1996). Soil provides the substrate for microbial growth, and the quantity and quality of litter regulate the rate of microbial decomposition (Crow et al., 2009; Scheibe et al., 2015).

In our study, *Proteobacteria* and *Actinobacteria* accounted for a large proportion of the soil microbial community in successional forests (Figures 2B,C). Compared with the early stage of succession, the abundance of *Proteobacteria* (r-strategist) decreased significantly and abundance of *Actinobacteria* (K-strategist) increased significantly in the middle stage (Supplementary Table S1). In addition, the ratio of (*Actinobacteria* + *Acidobacteria*) to (*Proteobacteria* + *Bacteroidetes*) increased significantly in the middle stage of succession (Supplementary Table S1). This phenomenon indicated that the middle forest succession leads to the transformation of bacterial community from the dominant r strategists to the dominant K-strategists (Yan et al., 2020). However, the r-strategists of bacteria did not show a decline trend in the late succession, which was not consistent with the Odum’s (1969) theory on ecosystem succession. We speculate that the high input of litterfall in the late forest succession provided more substrate for microbial decomposition, which may lead to a slight increase of r-strategists’ bacteria.

We did not find increasing trend of the relative abundance of *Acidobacteria* in successional forests (Supplementary Table S1). This result suggested that other environmental factors may play a more important role in microbial ecological strategies (Dai et al., 2021). The dominance of *Acidobacteria* may be related to the acidic soil of Dinghushan (pH value reached 3–4.5) (Liu et al., 2001), which promoted the growth of *Acidobacteria* (Dai et al., 2018). Fierer and Jackson (2006) found that soil pH value had important effects on soil bacterial community across different forest types. The negative correlation between the relative abundance of *Acidobacteria* and soil pH explained the highest relative abundance in the early stage of forest succession that had the lowest soil pH (Supplementary Figure S6).

Interestingly, fungal α-diversity decreased significantly in PBMF and MEBF compared with PMF, while fungal abundance increased significantly (Figure 1A), suggesting that microbial abundance and species diversity did not always respond the same way along the forest succession. It has been reported that the composition and abundance of soil fungal communities were affected by differences in litter composition and quality (Aponte et al., 2013; Prescott and Grayston, 2013). The decrease of fungi diversity in late-successional forest was also reported in previous studies (Zhang et al., 2018; Zhong et al., 2018). One possible explanation for this pattern is that certain fungal taxa within the dominant phyla are more efficient in utilizing available resources and have a competitive advantage in the nutrient-rich conditions of middle and late successional forests (Ghoul and Mitri, 2016; Chu et al., 2021). These dominant fungal taxa may exhibit higher growth rates and biomass accumulation, leading to an overall increase in fungal biomass despite a decrease in α-diversity. Meanwhile, less competitive or specialized fungal species may experience reduced abundance and diversity due to niche exclusion or resource competition with the dominant taxa (Pontarp and Petchey, 2018). Despite the α-diversity changed in middle and late successional forests, the ecological functions performed by the fungal communities may remain relatively unchanged. The relative abundance of fungal phyla may not change significantly because functionally similar species replace each other over time. In this case, even though fungi α-diversity decreases, the ecosystem may still maintain key ecological processes due to functional redundancy among the fungal species.

### 4.2 Variation of carbohydrate degradation potential of microbial community with succession

In the KEGG database, carbohydrate degradation genes changed significantly in different succession stages (Figure 3C), which was related to different plant communities formed by long-term forest succession. The carbohydrate degradation genes were significantly related to soil characteristics, litter characteristics, and microbial communities (Table 2), which was consistent with the research result (Ding et al., 2015). The correlation may be due to the difference in plant community structure and soil development degree during forest succession, which leads to the difference in microenvironment and soil nutrients (Lange et al., 2015), and then affects the microbial community structure and function (Wang et al., 2017).

This study revealed the environmental factors affecting the microbial community and functional diversity of soil organic carbon turnover. The significant changes in the carbohydrate degradation genes along the forest succession sequence indicated the existence of a succession distribution pattern of microbial functional diversity (Kelly et al., 2021). In general, the order of soil carbohydrate degradation is starch (the most mineralized), hemicellulose, pectin, cellulose, chitin, and lignin (Weil and Brady, 2016; Xie et al., 2022). Since we did not find lignin degradation genes in the KEGG database, we will not discuss.

In terrestrial ecosystems, litter decomposition is a key process of carbon exchange between plant carbon pool and soil carbon pool (Cusack et al., 2009). The gradual decomposition of litter returns carbon to soil and provides nutrients for plant and microbial growth (van der Heijden et al., 2008). In this study, we found that the amylase gene abundance was highest in PMF, and was positively correlated with litterfall and negatively correlated with TP (Figures 5, 6A). In general, the growth of microorganisms in the early stage of succession was mainly restricted by soil nutrient, but the large increase of litters could enhance the activity and growth of r-strategy microorganisms (Fontaine et al., 2003) and alleviate the low availability of soil nutrients. Dimassi et al. (2014) found that the priming effect of fresh organic matter was stronger in the soil with low nutrient content, but weaker in the soil with high nutrient content.

At the early stage of succession, the low soil phosphorus content led to tendency of r-strategy microorganisms to rapidly secrete enzymes to degrade litters to obtain phosphorus for growth and activity, which may lead to the rapid increase of soil amylase gene abundance. Compared with the early stage of succession, the increase of soil p content in the middle and late stages of succession led to the direct acquisition of phosphorus by microorganisms for the growth and activities in the soil, and the demand for nutrients from litter sources decreased, which may lead to the reduction of soil amylase gene abundance. R-strategy microorganisms tend to use fresh exogenous organic matter (Bremer and Kuikman, 1994). After substrate exhaustion, r-strategists die or become dormant because they are unable to use SOM (Fontaine et al., 2003). Moreover, the quantity of litter in the middle and late stage was significantly lower than that in the early stage, which may lead to the decrease of substrate content of r-strategy microorganisms, thus affecting the abundance of soil amylase gene. As the main contributor of amylase degradation, the relative abundance and relative contribution of *Proteobacteria* (r-strategist) reduced significantly in PBMF and MEBF compared with PMF, which further demonstrated our conclusion.

By contrast, gene abundance of hemicellulase, cellulase, and pectinase showed an upward trend with forest succession and had the highest degradation potential in MEBF (Figure 4), indicating the highest turnover rate of carbohydrates in the late-successional stage. We attributed it to the difference in soil carbon pool content. Previous studies reported that the late successional old-growth forests can still accumulate carbon in soil (Zhou et al., 2006a). Although the amount of exogenous input litter was significantly lower in MEBF than in PMF, the long-term forest succession resulted in significantly higher soil carbon content in MEBF than in PMF, which provided sufficient substrates for microorganisms to degrade soil refractory organic carbon in MEBF. Moreover, *Actinobacteria* and *Acidobacteria* (K-strategists) accounted for a large proportion of the contribution in the degradation of hemicellulose, cellulose, and pectin, and the gene abundance of hemicellulose, cellulase, and pectinase were mainly affected by SOC, TN, and SM (Figure 6). In our study, the strategy of the bacterial communities changed in the middle succession stage. The relative proportion of r strategists (*Proteobacteria*) decreased while the sum of the relative proportion of K-strategists (*Actinobacteria* and *Acidobacteria*) increased (Supplementary Table S1). Although the proportion of K-strategists did not change significantly in the late succession stage, the significant increase of total bacterial community elevated the degradation potential of K-strategists to a certain extent. Compared with r-strategists, which tend to use fresh exogenous organic matter (Bremer and Kuikman, 1994), K-strategists grow slowly and mainly use inexhaustible soil organic matter (Fontaine et al., 2003). Moreover, K-strategists can degrade the non-readily oxidation organic carbon to obtain nutrients required for its growth (Blagodatskaya et al., 2007), thus gradually dominating forest succession. When the abundance of K-strategists increases, the degradation of SOC will be promoted. Under the maximum SOC, SM, and TN values, the degradation potential of hemicellulase, cellulase, and pectinase genes was highest (Figures 5, 6), suggesting that the increase of soil SOC, SM, and TN may increase the K-strategy microbial biomass and activity, thus accelerating the decomposition of recalcitrant organic C.

## 5 Conclusion

Using metagenomic analysis, we investigated changes in soil bacterial and fungal community compositions in three subtropical successional forests and linked to the genetic potential for soil carbohydrate degradation. Soil bacterial and fungal communities showed increasing trends with forest succession, which were positively associated with soil and litter characteristics. Across all the successional stages, *Proteobacteria* and *Actinobacteria* dominated the bacterial community while *Ascomycota* maintained an absolute dominance in the fungal community. The strategy of bacteria transformed from r to K in middle succession stage, while the fungi strategy did not change during forest succession. However, the α-diversity of soil bacteria did not change during forest succession, and the α-diversity of soil fungi even decreased in the late-successional stage. Soil carbohydrate degradation functional genes such as hemicellulase, cellulase, and pectinase increased with forest succession, which were mainly affected by soil organic carbon, soil total nitrogen, and soil moisture, while the gene abundance of amylase was mainly affected by soil total phosphorus and litterfall. These findings emphasized the importance of soil microbial communities and their functions in forest soil carbon turnover and provided a better understanding of the mechanisms by which soil microbes and soil environments interactively drive soil carbon storage during subtropical forest succession.

## Data availability statement

The datasets presented in this study can be found in online repositories. The names of the repository/repositories and accession number(s) can be found in the article/Supplementary material.

## Author contributions

MH: Writing – original draft. SZ: Writing – review & editing. XX: Writing – review & editing. XW: Writing – review & editing. YS: Writing – review & editing. ZM: Writing – review & editing. DH: Writing – review & editing. JL: Writing – review & editing. DZ: Writing – review & editing. QD: Writing – review & editing.

## Footnotes

^1^https://github.com/OpenGene/fastp, version 0.20.0.

^2^https://github.com/voutcn/megahit, version 1.1.2.

^3^ http://metagene.cb.k.u-tokyo.ac.jp/

^4^http://www.bioinformatics.org/cd-hit/, version 4.6.1.

^5^http://soap.genomics.org.cn/, version 2.21.

^6^http://www.diamondsearch.org/index.php, version 0.8.35.

^7^http://www.genome.jp/keeg/, version 94.2.

^8^https://www.genome.jp/kegg/pathway.html, version 94.2.
