# Supplementary material for: Dynamics of soil microbial communities involved in carbon cycling along three successional forests in southern China
Source: Front Microbiol. 2024 Jan 15;14:1326057. doi: 10.3389/fmicb.2023.1326057 (PMC10822976; doi:10.3389/fmicb.2023.1326057)

**Supplementary material**

**
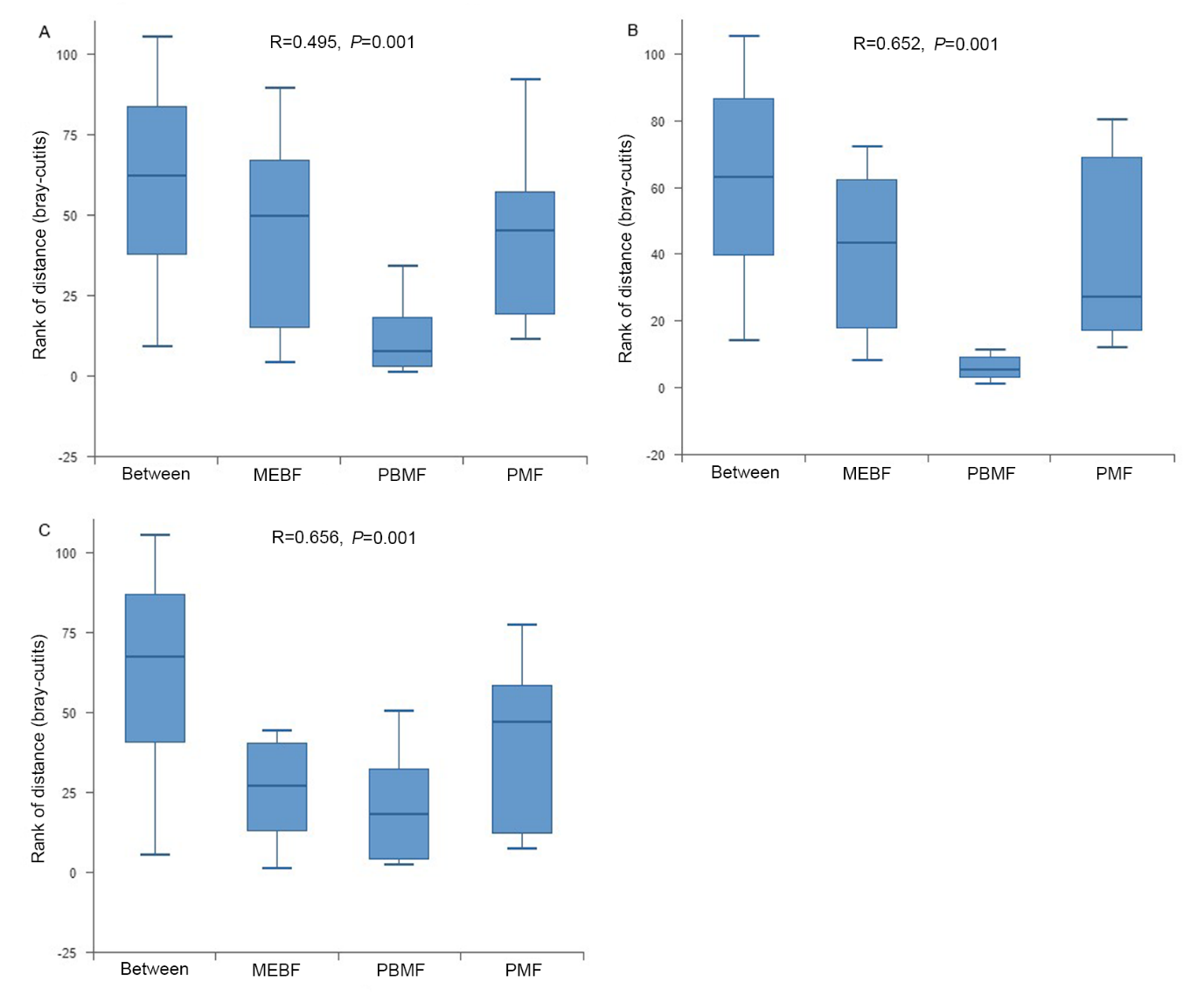
**

**Fig. S1.** Analysis of similarities between different groups of bacterial (A) and fungal (B) communities, and functional genes of carbohydrate degradation in microbial communities(C). R values range from -1 to +1; a positive R value indicates that the difference between groups is greater than the difference within groups. *P*-value less than or equal to 0.05 indicates the high reliability of this test. The permutation number is 999. PMF, *Pinus massoniana* forest; PBMF, pine and broadleaf mixed forest; MEBF, monsoon evergreen broadleaf forest.


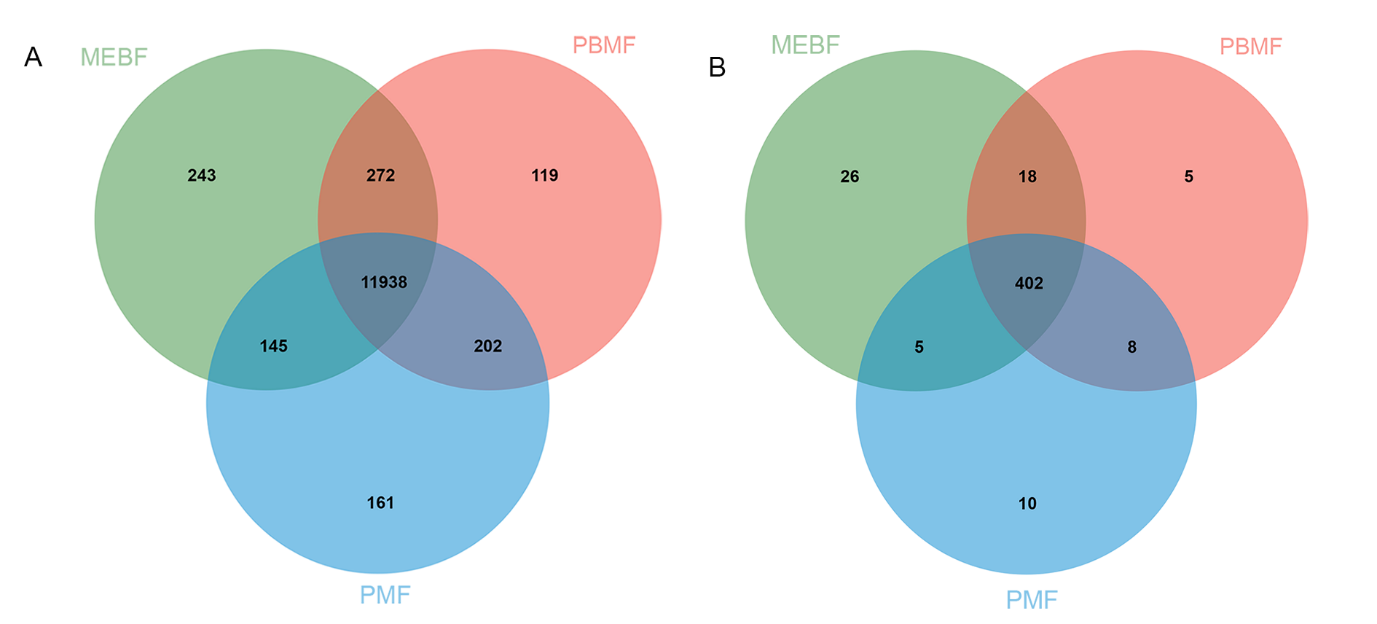


**Fig. S2.** [Venn diagram](https://www.sciencedirect.com/topics/earth-and-planetary-sciences/venn-diagram) of the number of shared and unique species among PMF, PBMF, and MEBF. PMF, *Pinus massoniana* forest; PBMF, pine and broadleaf mixed forest; MEBF, monsoon evergreen broadleaf forest.


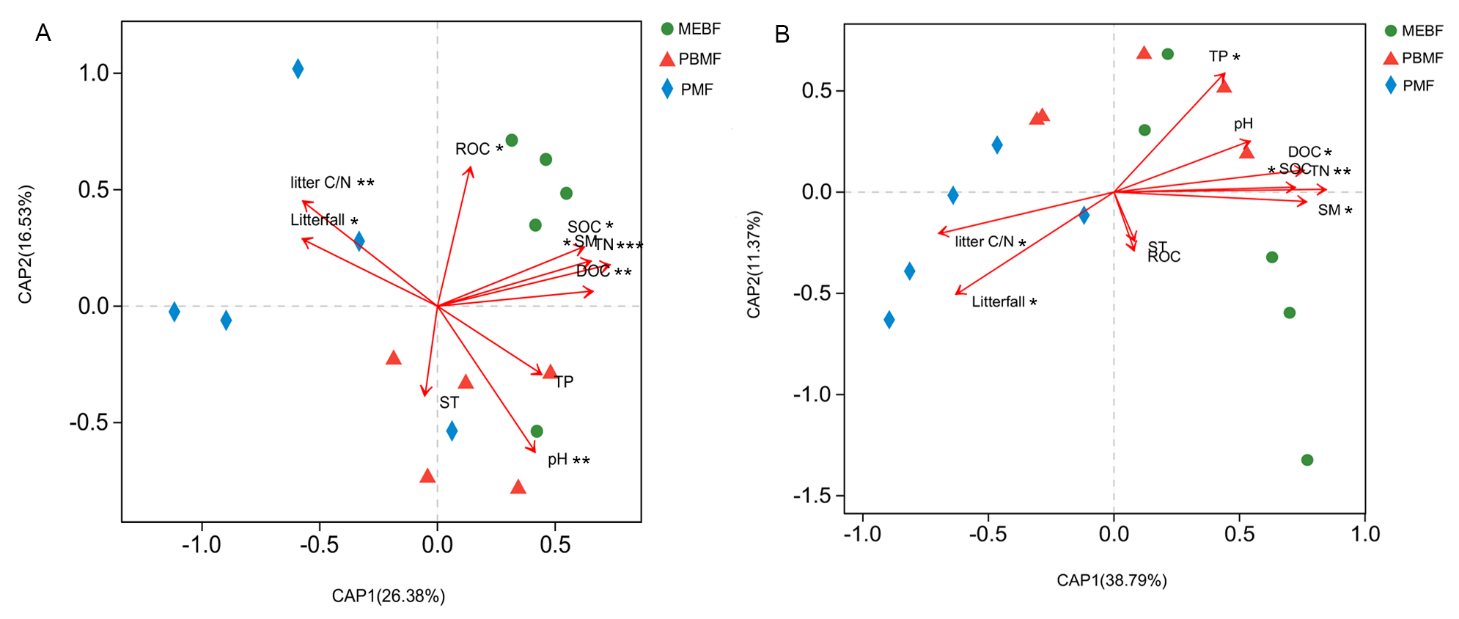


**Fig. S3.** Distance-based redundancy analysis (db-RDA) based on the Bray–Curtis distance of bacterial (A) and fungal (B) communities and soil physicochemical properties. SOC, total soil organic carbon; DOC, dissolved organic carbon; ROC, readily oxidised organic carbon; TN, total nitrogen; TP, total phosphorus; SM, soil moisture; ST, soil temperature; Litter C/N, the ratio of litter C to litter N. * indicates *P* ≤ 0.05; ** indicates *P* ≤ 0.01; *** indicates *P* ≤ 0.001.


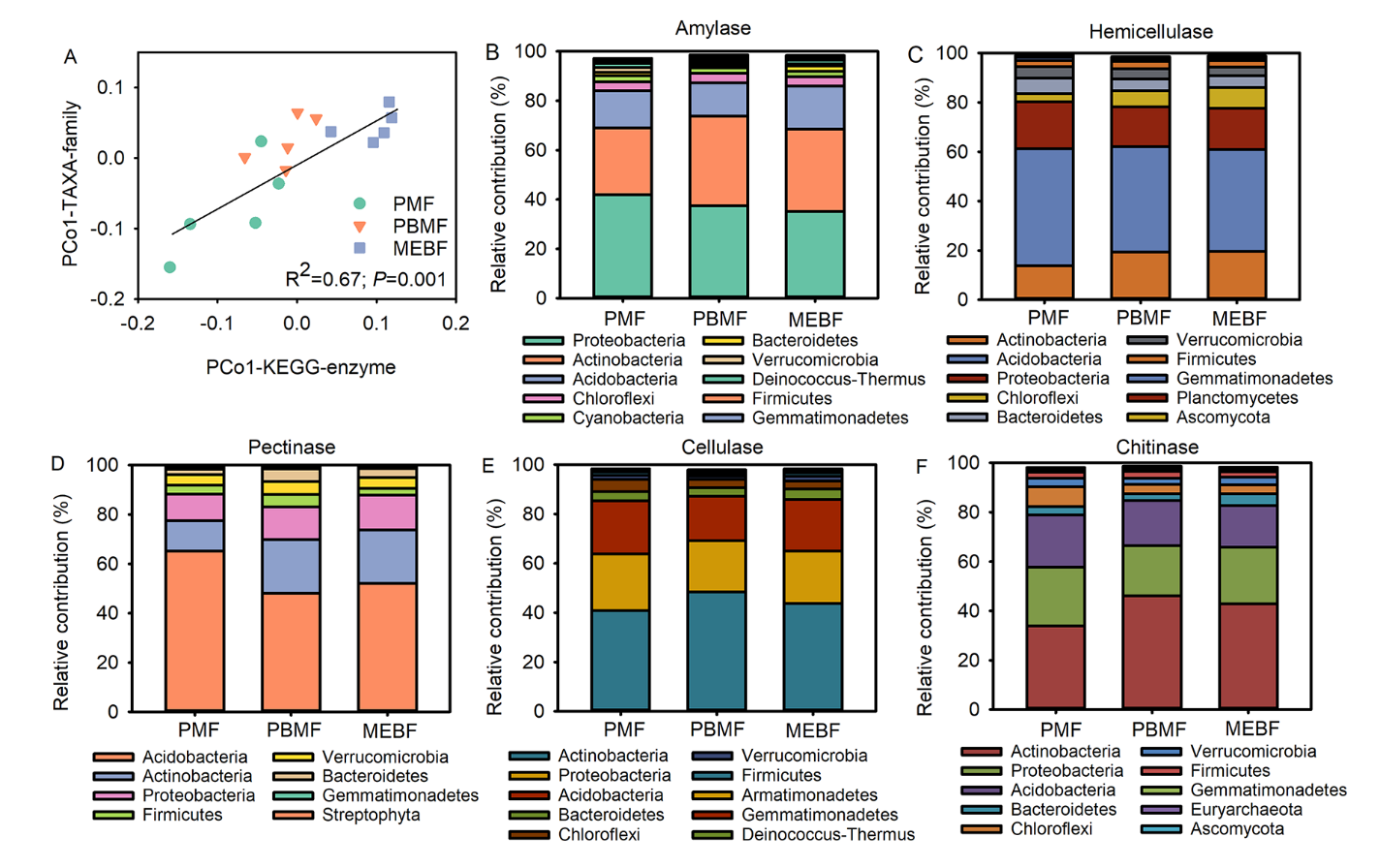


**Fig. S4.** Linear regression analysis of microbial communities and carbohydrate degradation genes β-diversity (A). Microbial communities and functional genes distances were calculated using the Bray–Curtis distance metrics. The relative contribution of dominant phylum in microbial communities to soil carbohydrate degradation in successional forests: (B) the contribution of microbial to amylase genes in soil starch degradation; (C) the contribution of microbial to hemicellulase genes in soil hemicellulose degradation; (D) the contribution of microbial to cellulase genes in soil cellulose degradation; (E) the contribution of microbial to pectinase genes in soil pectin degradation in successional forests; (F) the contribution of microbial to chitinase genes in soil chitin degradation. PMF, *Pinus massoniana* forest; PBMF, pine and broadleaf mixed forest; MEBF, monsoon evergreen broadleaf forest.


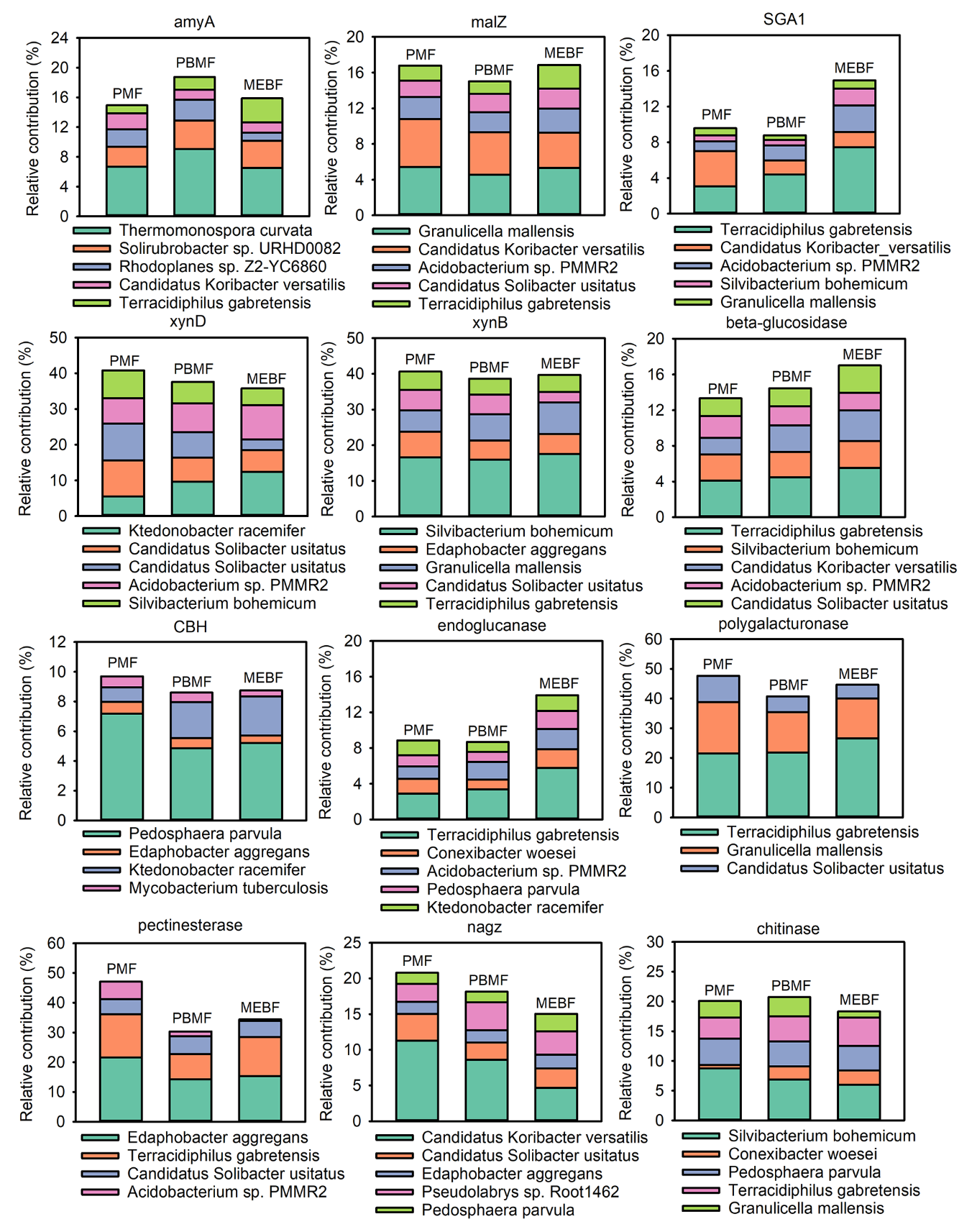


**Fig. S5.** The relative contribution of dominant species in microbial communities to soil carbohydrate degradation in successional forests. PMF, *Pinus massoniana* forest; PBMF, pine and broadleaf mixed forest; MEBF, monsoon evergreen broadleaf forest.


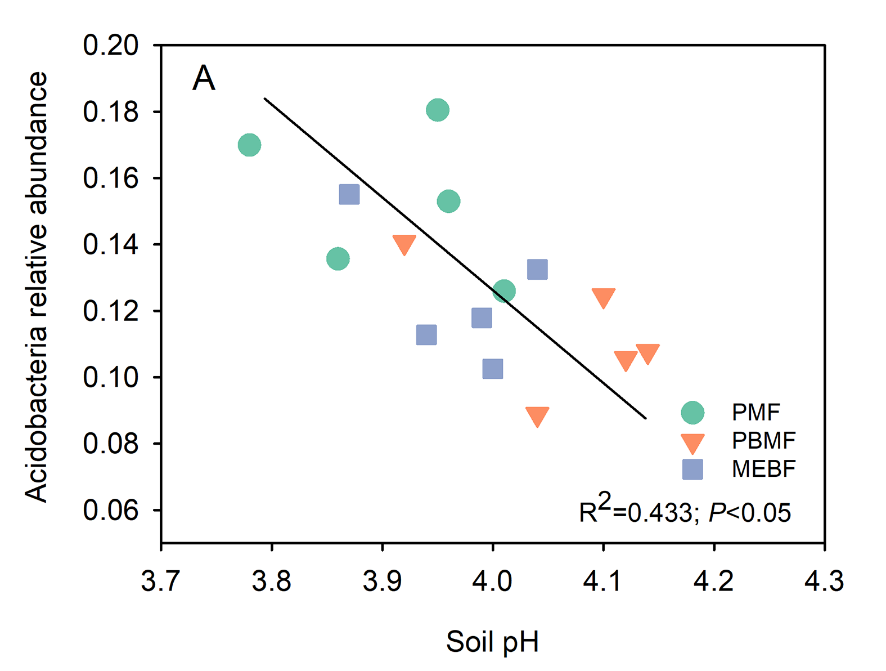


**Fig. S6.** The correlation between Acidobacteria relative abundance and soil pH. PMF, *Pinus massoniana* forest; PBMF, pine and broadleaf mixed forest; MEBF, monsoon evergreen broadleaf forest.

**Table S1** Relative abundance of bacteria and fungi at phylum level in successional forests. PMF, Pinus massoniana forest; PBMF, pine and broadleaf mixed forest; MEBF, monsoon evergreen broadleaf forest. Mean values ± standard deviations are given (n = 5). Different lowercase letters (in brackets) indicate a significant difference (*P* ≤ 0.05) among the forest types.

| Kingdom | phylum | PMF | PBMF | MEBF |
| --- | --- | --- | --- | --- |
| Bacteria | Proteobacteria | 41.63%±1.73% (b) | 36.06%±0.54% (a) | 36.91%±3.64% (a) |
|  | Actinobacteria | 28.38%±2.78% (a) | 41.51%±3.38% (b) | 38.45%±5.61% (b) |
|  | Acidobacteria | 15.3%±2.28% (b) | 11.36%±1.97% (a) | 12.42%±2.04% (ab) |
|  | Chloroflexi | 2.53%±0.59% (b) | 1.8%±0.12% (a) | 1.76%±0.49% (a) |
|  | Firmicutes | 1.85%±0.3% | 1.95%±0.17% | 1.95%±0.15% |
|  | Verrucomicrobia | 2.13%±0.47% (b) | 1.17%±0.34% (a) | 1.44%±0.45% (a) |
|  | Planctomycetes | 1.71%±0.22% (b) | 1.16%±0.12% (a) | 1.29%±0.31% (a) |
|  | Unclassified Bacteria | 1.42%±0.18% (b) | 1.21%±0.08% (a) | 1.15%±0.13% (a) |
|  | Cyanobacteria | 1.32%±0.12% (b) | 1.1%±0.04% (a) | 1.05%±0.11% (a) |
|  | Bacteroidetes | 0.85%±0.2% | 0.9%±0.19% | 1.24%±0.51% |
| (Actinobacteria + Acidobacteria) / (Proteobacteria + Bacteroidetes) | | 1.03%±0.06% (a) | 1.43%±0.07% (b) | 1.36%±0.28% (b) |
| Fungi | Ascomycota | 69.84%±2.67% | 71.24%±0.78% | 71.11%±3.07% |
|  | Basidiomycota | 23.18%±3.03% | 21.47%±1.21% | 21.78%±2.91% |
|  | Unclassified Fungi | 3.07%±0.16% | 3.27%±0.58% | 3.56%±0.49% |
|  | Chytridiomycota | 2.61%±0.45% | 2.59%±0.21% | 2.19%±0.75% |

**Table S2** Summary of the genome assembly. Clean reads: the number of sequences of clean reads; Clean bases: the total sequence length of clean reads; Percentage in raw reads: the percentage of clean reads to raw reads; Percentage in raw bases: the percentage of clean bases to raw bases. Contigs: the number of contigs sequences; Contigs bases: the total sequence length of contigs; N50: Sort contigs sequences by length and size, scanned length value of each contigs sequence one by one from the largest to the smallest and the accumulates. The longer the length of N50, the better the effect of assembly.

| Samples | Clean reads | Percentage in raw reads (%) | Clean base (bp) | Percentage in raw bases (%) | Contigs | Contigs bases (bp) | N50 (bp) |
| --- | --- | --- | --- | --- | --- | --- | --- |
| PMF1 | 9.05*10^7^ | 97.87 | 1.36*10^10^ | 97.62 | 1.41*10^6^ | 8.61*10^8^ | 626 |
| PMF2 | 9.25*10^7^ | 98.31 | 1.4*10^10^ | 98.17 | 1.44*10^6^ | 8.89*10^8^ | 627 |
| PMF3 | 8.71*10^7^ | 98.54 | 1.31*10^10^ | 98.42 | 1.46*10^6^ | 8.75*10^8^ | 618 |
| PMF4 | 8.57*10^7^ | 98.41 | 1.29*10^10^ | 98.27 | 1.19*10^6^ | 7.2*10^8^ | 627 |
| PMF5 | 9.33*10^7^ | 98.35 | 1.41*10^10^ | 98.22 | 1.35*10^6^ | 8.1*10^8^ | 613 |
| PBMF1 | 8.17*10^7^ | 98.37 | 1.23*10^10^ | 98.23 | 1.22*10^6^ | 7.97*10^8^ | 684 |
| PBMF2 | 9.51*10^7^ | 98.7 | 1.43*10^10^ | 98.6 | 1.5*10^6^ | 9.17*10^8^ | 630 |
| PBMF3 | 8.95*10^7^ | 98.69 | 1.35*10^10^ | 98.56 | 1.46*10^6^ | 9.13*10^8^ | 647 |
| PBMF4 | 8.58*10^7^ | 98.74 | 1.29*10^10^ | 98.64 | 1.43*10^6^ | 9.31*10^8^ | 683 |
| PBMF5 | 8.66*10^7^ | 98.63 | 1.31*10^10^ | 98.52 | 1.35*10^6^ | 8.03*10^8^ | 613 |
| MEBF1 | 9.12*10^7^ | 96.58 | 1.38*10^10^ | 96.46 | 1.5*10^6^ | 1.03*10^9^ | 723 |
| MEBF2 | 8.29*10^7^ | 97.78 | 1.25*10^10^ | 97.6 | 1.13*10^6^ | 7.97*10^8^ | 745 |
| MEBF3 | 1*10^8^ | 97.67 | 1.51*10^10^ | 97.5 | 1.6*10^6^ | 1.05*10^9^ | 676 |
| MEBF4 | 9.87*10^7^ | 96.04 | 1.49*10^10^ | 95.91 | 1.6*10^6^ | 1.01*10^9^ | 639 |
| MEBF5 | 9.91*10^7^ | 97.71 | 1.49*10^10^ | 97.56 | 1.5*10^6^ | 1.02*10^9^ | 722 |

**Table S3** Detailed information of microbial functional genes for soil carbohydrates degradation based on the KEGG database.

| Key enzyme | EC number | Substrate category | Reference |
| --- | --- | --- | --- |
| α-amylase | 3.2.1.1 | Starch | (Bertoldo and Antranikian, 2002) |
| α-glucosidase | 3.2.1.20 | Starch |  |
| Glucan 1,4-α-glucosidase | 3.2.1.3 | Starch |  |
| α-L-arabinofuranosidase | 3.2.1.55 | Hemicellulose | (Perez et al., 2002) (Shallom et al., 2003) |
| β-xylosidase | 3.2.1.37 | Hemicellulose |  |
| Endo-1,4-β-xylanases | 3.2.1.8 | Hemicellulose |  |
| Endopolygalacturonase | 3.2.1.15 | Pectin | (Xue et al., 2005)  (Cline et al., 2005) |
| Galacturan 1,4-α-galacturonidase | 3.2.1.67 | Pectin |  |
| Pectate lyase | 4.2.2.2 | Pectin |  |
| Pectin lyase | 4.2.2.10 | Pectin |  |
| Pectinesterase | 3.1.1.11 | Pectin |  |
| β-glucosidase | 3.2.1.21 | Cellulose | (Perez et al., 2002)  (McMahon et al., 2006) |
| Cellulose 1,4-β-cellobiosidase | 3.2.1.91 | Cellulose |  |
| Endocellulase | 3.2.1.4 | Cellulose |  |
| α-N-acetylglucosaminidase | 3.2.1.50 | Chitin | (Krsek et al., 2003) |
| β-N-acetylhexosaminidase | 3.2.1.52 | Chitin |  |
| Chitin deacetylase | 3.5.1.41 | Chitin |  |
| Endochitinase | 3.2.1.14 | Chitin |  |
| Laccase | 1.10.3.2 | Lignin | (Perez et al., 2002)  (McMahon et al., 2007) |
| Lignin peroxidase | 1.11.1.14 | Lignin |  |
| Manganese peroxidase | 1.11.1.13 | Lignin |  |

**Table S4** Carbohydrate degradation genes abundance in successional forests. PMF, *Pinus massoniana* forest; PBMF, pine and broadleaf mixed forest; MEBF, monsoon evergreen broadleaf forest. Mean values ± standard deviations are given (*n* = 5). Different lowercase letters (in brackets) indicate a significant difference (*P* ≤ 0.05) among the forest types.

| Substrate category | Gene | EC number | PMF | PBMF | MEBF |
| --- | --- | --- | --- | --- | --- |
| Starch | amyA | 3.2.1.1 | 716.43±31.82 (c) | 635.83±22.86 (a) | 673±11.43 (b) |
| Starch | malZ | 3.2.1.20 | 563.41±14.43 (c) | 512.81±18.49 (a) | 538.3±10.61 (b) |
| Starch | SGA1 | 3.2.1.3 | 357.66±22.53 (b) | 311.17±12.74 (a) | 329.09±14.87 (a) |
| Hemicellulose | xynD | 3.2.1.55 | 257.94±11.53 (a) | 309.54±23.02 (b) | 327.69±32.37 (b) |
| Hemicellulose | xynB | 3.2.1.37 | 180.49±14.19 (a) | 175.39±14.04 (a) | 198.17±9.58 (b) |
| Pectin | polygalacturonase | 3.2.1.15 | 43.79±8.89 (a) | 53.1±10.48 (a) | 99.49±17.42 (b) |
| Pectin | E3.2.1.67 | 3.2.1.67 | 0.35±0.25 (a) | 0.7±0.37 (a) | 1.57±0.71 (b) |
| Pectin | pectinesterase | 3.1.1.11 | 16.2±4.79 (a) | 23.65±6.58 (ab) | 31.27±7.51 (b) |
| Pectin | pel | 4.2.2.2 | 2.9±1.5 | 4.04±1.8 | 3.98±1.53 |
| Cellulose | beta-glucosidase | 3.2.1.21 | 1827.61±14.64 (a) | 1837.95±6.58 (a) | 1929.16±32.09 (b) |
| Cellulose | CBH | 3.2.1.91 | 26.91±4.61 (a) | 49.98±3.79 (b) | 57.88±4.49 (c) |
| Cellulose | endoglucanase | 3.2.1.4 | 542.09±12.98 (a) | 558.97±16.42 (ab) | 577.08±19.24 (b) |
| Chitin | nagZ | 3.2.1.52 | 673.44±18.86 | 652.12±34.16 | 659.95±13.64 |
| Chitin | chitin deacetylase | 3.5.1.41 | 2.38±0.87 | 1.63±0.74 | 1.93±1.34 |
| Chitin | chitinase | 3.2.1.14 | 332.69±9.38 | 319.1±15.57 | 329.96±24.95 |

**Reference**

Bertoldo, C., Antranikian, G., 2002. Starch-hydrolyzing enzymes from thermophilic archaea and bacteria. Curr. Opin. Cell Biol. 6, 151-160. <https://doi.org/>10.1016/S1367-5931(02)00311-3.

Cline, L.C., Zak, D.R., 2015. Soil microbial communities are shaped by plant-driven changes in resource availability during secondary succession. Ecology 96, 3374-3385. <https://doi.org/>10.1890/15-0184.1.

Krsek, M., Wellington, E.M.H., 2001. Assessment of chitin decomposer diversity within an upland grassland. Anton Leeuw Int J G 79, 261-267. <https://doi.org/>10.1023/A:1012043401168.

McMahon, A.M., Doyle, E.M., Brooks, S., O'Connor, K.E., 2007. Biochemical characterisation of the coexisting tyrosinase and laccase in the soil bacterium Pseudomonas putida F6. Enzyme Microb. Technol. 40, 1435-1441. <https://doi.org/>10.1016/j.enzmictec.2006.10.020.

Perez, J., Munoz-Dorado, J., de la Rubia, T., Martinez, J., 2002. Biodegradation and biological treatments of cellulose, hemicellulose and lignin: An overview. Int. Microbiol. 5, 53-63. <https://doi.org/>10.1007/s10123-002-0062-3.

Shallom, D., Shoham, Y., 2003. Microbial hemicellulases. Curr. Opin. Cell Biol. 6, 219-228. <https://doi.org/>10.1016/S1369-5274(03)00056-0, 2003.

van den Brink, J., de Vries, R.P., 2011. Fungal enzyme sets for plant polysaccharide degradation. Appl. Microbiol. Biotechnol. 91, 1477-1492. <https://doi.org/>10.1007/s00253-011-3473-2.

Xue, C.H., Zhang, Y.Q., Jie, L.Z., Jun, L.Z., 2005. Recent development of pectin and pectolytic enzyme. J. Food Sci. Biotechnol. 24, 94-99. (in Chinese with English abstract).
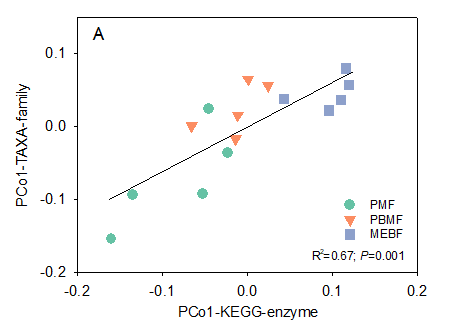

Supplement: Supplementary file 1 [file Data_Sheet_1.docx]
